# Genetic polymorphisms in interleukin-1β (rs1143634) and interleukin-8 (rs4073) are associated with survival after resection of intrahepatic cholangiocarcinoma

**DOI:** 10.1038/s41598-023-39487-7

**Published:** 2023-07-28

**Authors:** Isabella Lurje, Nadine Therese Gaisa, Edgar Dahl, Ruth Knüchel, Pavel Strnad, Christian Trautwein, Frank Tacke, Ulf Peter Neumann, Zoltan Czigany, Georg Lurje

**Affiliations:** 1 Department of Hepatology and Gastroenterology, Campus Charité Mitte | Campus Virchow-Klinikum, Charité-Universitätsmedizin Berlin, Berlin, Germany; 2 Department of Surgery and Transplantation, University Hospital RWTH Aachen, Aachen, Germany; 3 Institute of Pathology, University Hospital RWTH Aachen, Aachen, Germany; 4 Department of Surgery, Campus Charité Mitte | Campus Virchow-Klinikum, Charité-Universitätsmedizin Berlin, Augustenburger Platz 1, 13353 Berlin, Germany; 5 Department of Internal Medicine III, University Hospital RWTH Aachen, Aachen, Germany

**Keywords:** Cancer microenvironment, Liver cancer, Prognostic markers

## Abstract

Intrahepatic cholangiocarcinoma (iCCA) is a rare, understudied primary hepatic malignancy with dismal outcomes. Aiming to identify prognostically relevant single-nucleotide polymorphisms, we analyzed 11 genetic variants with a role in tumor-promoting inflammation (*VEGF, EGF, EGFR, IL-1B, IL-6, CXCL8 (IL-8), IL-10, CXCR1, HIF1A* and *PTGS2 (COX-2)* genes) and their association with disease-free (DFS) and overall survival (OS) in patients undergoing curative-intent surgery for iCCA. Genomic DNA was isolated from 112 patients (64 female, 48 male) with iCCA. Germline polymorphisms were analyzed with polymerase chain reaction-restriction fragment length polymorphism protocols. The *IL-1B* +3954 C/C (73/112, hazard ratio (HR) = 1.735, *p* = 0.012) and the *IL-8* -251 T/A or A/A (53/112 and 16/112, HR = 2.001 and 1.1777, p = 0.026) genotypes were associated with shorter OS in univariable and multivariable analysis. The *IL-1B* +3954 polymorphism was also associated with shorter DFS (HR = 1.983, p = 0.012), but this effect was not sustained in the multivariable model. A genetic risk model of 0, 1 and 2 unfavorable alleles was established and confirmed in multivariable analysis. This study supports the prognostic role of the *IL-1B* C+3954T and the *IL-8* T-251A variant as outcome markers in iCCA patients, identifying patient subgroups at higher risk for dismal clinical outcomes.

## Introduction

Cholangiocarcinoma (CCA) is one of the most aggressive gastrointestinal cancers with a rising worldwide incidence over the last decade^1^. Despite the improvement of surgical techniques and palliative regimens, therapeutic options remain limited and outcomes are dismal^2^. From the anatomical and surgical perspective, CCA can be classified into intrahepatic (iCCA), perihilar (pCCA) and distal (dCCA) disease. While iCCAs arise above the second-order bile ducts, pCCAs originate above the cystic duct and below the second-order bile ducts and dCCAs below the cystic duct, and, as a consequence, surgical approaches differ significantly between entities^3,4^.

Intrahepatic CCA (iCCA) represents approximately 10–20% of CCA and is an understudied tumor entity^3^. Predisposing factors include chronic biliary inflammation such as primary sclerosing cholangitis, as well as cholelithiasis and liver cirrhosis, but more general risk factors such as type 2 diabetes and smoking have been described as well^5^. To date, surgery represents the only curative treatment for iCCA, with dismal survival rates of 20%-35% after 5 years^6^. Clinico-pathological characteristics such as lymphovascular invasion and poor differentiation remain the best-studied prognostic factors, which, however, have a limited value for the preoperative identification of patients at risk for poor postoperative outcomes^6,7^. Therefore, finding prognostic markers as an adjunct to traditional staging systems may facilitate the selection of patients who require additional or a more aggressive adjuvant treatment approaches and a closer oncological follow-up^8^.

The tumor microenvironment (TME) of iCCA is abundant in mediator responses that drive tumor growth and invasion while abrogating anti-tumor immune responses including antigen presentation and infiltration of activated cytotoxic T cells. Typically, a prominent desmoplastic reaction with a proliferation of cancer-associated fibroblasts (CAF) and an infiltration of immunosuppressive myeloid and lymphoid populations are present^9^. Neoangiogenesis, an essential prerequisite for tumor growth, is driven by vascular endothelial growth factor (VEGF) and supported by monocytes^10,11^. Furthermore, infiltrating immune cells convey tolerogenic effects that abrogate efficient antigen cross-presentation and cytotoxic T cell antitumor activity^12,13^.

We hypothesized that functional gene polymorphisms encoding for proteins that are critically involved in the tumor microenvironment may have prognostic value in iCCA by altering the systemic and local concentration of mediators relevant for the TME. We hypothesized that an altered expression of proteins involved in the attraction of suppressive myeloid populations such as tumor-associated neutrophils (TANs) and tumor-associated macrophages (TAM)—like interleukin (IL)-1β or Hypoxia-inducible factor (HIF)-1α—may impact prognosis in these patients^14,15^. Further selected single nucleotide polymorphism (SNP) candidates were in genes encoding for mediators in VEGF-dependent and independent angiogenesis (VEGF, IL-8)^16,17^. Thus, we analysed 11 polymorphisms in ten genes with a role in tumor inflammation and tumor-related immunosuppression to identify patient subgroups with dismal oncological and overall outcome after surgical resection of cholangiocarcinoma.

## Patients and methods

### Study population

In this retrospective single-center study, data of *N* = 112 consecutive patients with localized iCCA undergoing curative-intent surgery at the University Hospital RWTH Aachen were analysed. Clinico-pathological and survival data for this study was obtained from a prospectively managed institutional database spanning 2010–2019. A part of the included cohort had previously been analyzed to determine the efficacy of the surgical ALPPS technique for iCCA^18^, the prognostic role of pathological factors^7^ and small nerve fibers^19^. Patients with mixed hepatocellular carcinoma (HCC)-CCA histology or neuroendocrine tumor differentiation were not included in the analysis, nor were pCCA and dCCAs, due to different tumor biology, prognostic factors, and surgical treatment. Patients with extrahepatic or metastatic disease were excluded, as well. An overview of the selection criteria is provided in Supplementary Fig. 1. A senior hepatobiliary pathologist (NTG) reviewed the tumor histology. Patient material for genotyping was provided by the institutional biobank (RWTH-cBMB) and the Department of Pathology (NTG, ED, RKC). This study was approved by the institutional review board of the RWTH Aachen University (EK 360/15, EK 173/06) and conducted in accordance with good clinical practice guidelines and the current Declaration of Helsinki. For this study informed consent has been waived by Institutional review board of the RWTH Aachen University, EK 360/15, EK 173/06 due to the anonymity and retrospective nature of the study. An ex-ante sample size calculation was not performed due to the hypothesis-generating, exploratory study design.

### Staging and surgical technique

Preoperative work-up included appropriate cross-sectional imaging to rule out distant metastases and CT or magnetic resonance imaging (MRI) of the liver to visualize hilar vessel invasion and, if necessary, endoscopic retrograde cholangiopancreatography (ERCP) or magnetic resonance cholangiopancreatography (MRCP) to assess hilar disease extent. Patients with suspected metastatic disease on conventional imaging underwent positron emission tomography (PET)-CT. In cases of insufficient estimated future liver remnant on liver volumetry, portal vein embolization (PVE) and, if necessary, ALPPS, were employed to allow right-sided hepatectomy. Indication for surgical resection was based on the recommendation of senior hepatobiliary surgical staff and approved by the local multidisciplinary tumor board. Depending on tumor extent, the resection volume ranged from atypical/non-anatomical to extended resections^7^.

An experienced board-certified staff pathologist performed the routine histopathological work-up and reported tumor type, histopathological grading and staging, loco-regional lymph node metastasis, resection margins and vessel invasion.

### SNP selection

The polymorphisms were selected in a pathway-centered approach, with the aim of selecting genes involved in tumor-associated inflammation and neovascularization, as well as tumor immune suppression (Supplementary Table 1). The following prerequisites were set: (a) that the gene is a part of a pathway involved in tumor-associated inflammation and tumor immunosuppression, (b) that the respective polymorphism is well-documented and confers a biological effect, and (c) that the frequency of the polymorphism is sufficient to enable a statistically meaningful association with clinical outcomes. In line with previous studies, this was estimated to be the case if at least 15% of the general population carry the minor allele of the genetic variant^20^. A total of 11 SNPs in ten genes were selected, including *VEGF, Epidermal Growth Factor* (*EGF*), *EGF-Receptor* (*EGF-R*), *IL-1B, IL-6, C-X-C motif chemokine ligand* (*CXCL*)*8 *(*IL-8*), *IL-10, CXC chemokine receptor *(*CXCR*)*1, HIF1A* *and Prostaglandin-Endoperoxide Synthase* (*PTGS2, COX2*) (Table 1).

**Table 1:** Patient characteristics in association with disease-free and overall survival in iCCA.

	n = 112	Median DFS (95%*CI*)	*HR*	*p*^†^	Median OS (95%CI)	*HR*	*p*^†^
Sex							
Male	48 (42.9)	11 (3.799–18.201)		.984	19 (14.026–23.974)		.332
Female	64 (57.1)	12 (8.055–15.945)			22 (8.991–35.009)		
Age, years							
≤ 65	50 (46.3)	10 (4.826–15.174)		.121	20 (7.675–32.325)		.189
> 65	58 (53.7)	16 (5.245–26.755)			21 (10.767–31.233)		
BMI, kg/m^2^							
≤ 25	54 (50.0)	12 (8.834–15.166)		.456	25 (14.655–35.345)		.438
> 25	54 (50.0)	12 (5.069–18.931)			18 (10.975–25.025)		
PVE							
No	101 (90.2)	12 (8.371–15.629)		.293	19 (5.054–32.946)		.556
Yes	11 (9.8)	27 (2.564–51.436)			21 (12.068–29.932)		
EBD							
No	91 (84.3)	30 (7.940–52.060)		.147	22 (13.540–30.460)		.567
Yes	17 (15.7)	10 (7.281–12.719)			13 (7.697–18.303)		
PBD							
No	107 (99.1)	n.a		.922	21 (15.483–26.517)	1	**.000**
Yes	1 (0.9)	0 (n.a.)			0 (n.a.)	26.796 (2.998–239.470)	
Albumin, g/l							
≤ 42	35 (32.7)	8 (3.261–12.739)		.145	13 (5.233–20.767)		.093
> 42	72 (67.3)	13 (6.737–19.263)			28 (17.830–38.170)		
AST, U/l							
≤ 40	63 (58.9)	13 (5.926–20.074)		.646	20 (15.442–24.558)		.863
> 40	44 (41.1)	10 (7.171–12.829)			25 (13.677–36.323)		
ALT, U/l							
≤ 40	56 (52.3)	16 (8.345–23.655)		.473	21 (9.721–32.279)		.763
> 40	51 (47.7)	8 (3.753–12.247)			20 (9.671–30.329)		
GGT, U/l							
≤ 100	45 (42.1)	13 (3.341–22.659)		.225	25 (15.125–34.875)		.080
> 100	62 (57.9)	11 (6.586–15.414)			19 (12.674–25.326)		
Bilirubin, mg/dl							
≤ 1	85 (79.4)	12 (6.830–17.170)		.909	20 (15.746–24.254)		.986
> 1	22 (20.6)	8 (1.598–14.402)			22 (4.523–39.477)		
Alkaline phosphatase, U/l							
≤ 100	37 (34.6)	18 (8.585–27.415)		.076	28 (n.a.)	1	**.018**
> 100	70 (65.4)	10 (7.035–12.965)			18 (9.172–26.828)	1.854 (1.096–3.137)	
Platelet count, 1/nl							
≤ 200	27 (25.2)	8 (1.038–14.962)		.465	18 (3.400–32.600)		.051
> 200	80 (74.8)	12 (4.556–19.444)			25 (13.197–36.803)		
INR							
≤ 1	57 (53.3)	13 (5.176–20.824)		.200	25 (15.593–34.407)		.125
> 1	50 (46.7)	11 (7.227–14.773)			14 (7.408–20.592)		
Hemoglobin, g/dl							
≤ 12	29 (27.1)	11 (1.866–20.134)		.078	6 (0.000–13.911)	1	**.000**
> 12	78 (72.9)	13 (5.490–20.510)			25 (4.034–45.966)	0.426 (0.262-0.690)	
CRP, mg/l							
≤ 10	56 (52.3)	13 (6.215–19.785)		.133	28 (4.327–51.673)	1	**.008**
> 10	51 (47.7)	10 (5.677–14.323)			14 (3.779–24.221)	1.845 (1.155–2.946)	
Knife-to-skin time, min							
≤ 300	63 (58.3)	13 (6.240–19.760)		.168	22 (14.682–29.318)		.307
> 300	45 (41.7)	8 (2.638–13.362)			20 (5.522–34.478)		
Blood transfusions							
No	71 (65.7)	15 (7.864–22.136)	1	**.025**	25 (14.793–35.207)	1	**.008**
Yes	37 (34.3)	8 (1.237–14.763)	1.756 (1.054–2.926)		10 (0.000–20.479)	1.866 (1.163–2.993)	
FFP							
No	64 (59.3)	12 (6.499–17.501)		.344	25 (16.927–33.073)		.100
Yes	44 (40.7)	12 (7.183–16.817)			14 (4.115–23.885)		
R1 status							
R0	80 (74.1)	12 (5.259–18.741)		.068	29 (10.817–47.183)	2.136 (1.261–3.618)	**.003**
R1/Rx	23 (21.3)	8 (6.200–9.800)			9 (3.131–14.869)		
MVI							
No	63 (63.0)	13 (0.000–28.857)	1	**.006**	25 (12.755–37.245)	1	**.000**
Yes	37 (37.0)	9 (6.106–11.894)	2.076 (1.250–3.447)		20 (14.607–25.393)	1.339 (0.818–2.192)	
LVI							
No	71 (72.4)	12 (4.656–19.344)	1	**.015**	40 (16.205–63.795)	1	**.000**
Yes	27 (27.6)	4 (0.000–10.174)	2.022 (1.115–3.668)		4 (2.735–5.265)	4.274 (2.536–7.204)	
Tumor grading							
G1/G2	61 (56.5)	12 (6.592–17.408)		.417	28 (15.116–40.884)		.120
G3/G4	31 (28.7)	10 (5.852–14.148)			12 (2.726–21.274)		
Tumor stage UICC							
I/II	53 (55.7)	18 (9.317–26.683)	1	**.000**	50 (28.949–71.051)	1	**.000**
III/IV	42 (44.2)	7 (3.635–10.365)	2.509 (1.462–4.305)		9 (4.469–13.531)	3.463 (2.085–5.753)	
pT category							
pT1–2	98 (90.7)	12 (7.233–16.767)		.237	22 (13.909–30.091)	1	**.009**
pT3–4	9 (8.3)	9 (1.791–16.209)			9 (6.228–11.772)	2.571 (1.218–5.428)	
N category							
pN0	55 (57.9)	18 (9.534–26.466)	2.017 (1.172–3.469)	**.008**	50 (34.936)	1	**.000**
pN1	40 (42.1)	6 (1.545–10.455)			7 (1.687–12.313)	3.129 (1.885–5.196)	
Length of ICU stay, days							
≤ 1	70 (64.8)	13 (8.394–17.606)		.289	29 (12.239–45.761)	1	**.007**
> 1	38 (35.2)	8 (4.894–11.106)			7 (0.000–15.359)	1.877 (1.169–3.012)	
Length of hospitalization, days							
≤ 14	60 (55.6)	12 (6.058–17.942)		.279	29 (4.196–53.804)	1	**.003**
> 14	48 (44.4)	8 (3.550–12.450)			13 (1.965–24.035)	1.970 (1.238–3.134)	
**CCI**							
≤ 40	72 (64.3)	12 (6.413–17.587)		.167	28 (6.693–49.307)	1	**.006**
> 40	35 (31.3)	8 (4.656–11.344)			13 (.000–27.602)	1.937 (1.195–3.140)	
Adjuvant therapy							
No	51 (45.5)	39 (n.a.)	1	**.000**	22 (12.882–31.118)		.425
Yes	56 (50.0)	7 (5.742–8.258)	3.320 (1.893–5.820)		20 (11.527–28.473)		
Tumor recurrence							
No	42 (37.5)	n.a		n.a	25 (0.000–86.963)		.134
Yes	65 (58.0)	n.a			20 (16.995–23.005)		

*ALT* alanine aminotransferase, *AST* aspartate aminotransferase, *BMI* body mass index, *CCA* cholangiocarcinoma, *CCI* comprehensive complication index, *CI* confidence interval, *CRP* C-reactive protein, *DFS* disease-free survival, *EBD* endoscopic biliary drainage, *FFP* fresh frozen plasma, *GGT* gamma-glutamyl transferase, *HR* hazard ratio, *ICU* intensive care unit, *INR* international normalized ratio, *LVI* lymphovascular invasion, *MVI* microvascular invasion, *OS* overall survival, *PBD* percutaneous biliary drainage, *PVE* portal venous embolization, *UICC* union internationale contre le cancer.

^†^Based on log-rank test.

Significant values are in bold.

### Genotyping

Formalin-fixed paraffin-embedded non-tumor tissues were collected and the QIAamp DNA extraction kit (Qiagen, CA, Valencia, USA) was used to extract genomic DNA according to the manufacturer’s protocol. DNA quality and content was analysed photometrically (NanoDrop, Thermo Fisher, MA, USA). The polymerase chain reaction–restriction fragment length polymorphism (PCR–RFLP) technique was employed for genotyping, as previously reported^21,22^. The SNP region was amplified in 35 PCR cycles with forward- and reverse-primers, which were designed with the National Library of Medicine gene database and then controlled for alternative binding sites with the NCBI/National Center for Biotechnology Information primer blast function. The amplicon was digested with appropriate DNA restriction endonucleases specific for the SNP regions (New England Biolabs, MA, USA) (Supplementary Table 2). Then, the reaction products were separated on a 4% agarose gel at 120 mV for 60 min and visualized (GelDoc, Bio-Rad Laboratories GmbH, Feldkirchen, Germany) together with a 50 base pair DNA ladder. Based on the visualized fragment length and count, it was determined whether the region targeted by the restriction nucleases was digested. Appropriate positive (homocygous genotype of the smaller digested fragment) and negative controls (mastermix plus restriction enzyme, without DNA) were included on the gels. For quality control, 10% of positive and negative samples were randomly selected and re-genotyped with a genotype concordance ≥ 98%.

### Endpoints and statistical analysis

Disease-free survival (DFS) was defined as the period between surgery and first recurrence and patients were censored if they died without recurring. Overall survival (OS) was defined as the period between surgery and death without censoring for perioperative mortality. Individuals lost to follow-up were censored at the time of last patient contact. Differences in categorical variables were evaluated using two-tailed Fisher’s exact test and chi-squared test, in continuous variables with the *Mann–*Whitney *U* test. Kruskal–Wallis test was used to compare non-parametric variables with more than two groups. Continuous clinical variables were dichotomized at the median for the categorical presentation in the survival analysis. Differences in DFS and OS between genotypes were assessed with Kaplan–Meier analysis and the log-rank test for group comparison. For SNPs with a homozygous minor allele frequency < 10% in the study population, a dominant model was employed to test associations between genotypes and clinical outcome. Otherwise, a codominant or additive model was used. Uni- and multivariable Cox proportional hazard models were employed to analyze the association of factors with DFS and OS. Hazard ratios (HR) were presented with 95% confidence intervals (CI). Due to the large number of examined variables, only variables significant in the univariable analyses were included in the multivariable analyses, with an exclusion of parameters with potential collinearity. The level of significance was set to *p* < 0.05. Analyses were performed with SPSS Statistics (v23, IBM Corp., Armonk, NY, USA).

### Statement of ethics

This research complies with the guidelines for human studies and was conducted ethically in accordance with the World Medical Association Declaration of Helsinki.

### Study approval statement

This study protocol was reviewed and approved by institutional review board of the RWTH Aachen University (EK 360/15, EK 173/06). For this study informed consent has been waived by Institutional review board of the RWTH Aachen University, EK 360/15, EK 173/06 due to the anonymity and retrospective nature of the study.

## Results

### Clinical and histopathological characteristics and clinical outcome

Of the 112 patients undergoing curative-intent surgery for localized iCCA, 48 (43%) patients were male and 64 (57%) were female. Median age in this cohort at the time of surgical resection was 65 (range: 31–87) years. A total of 57 (51%) of patients received adjuvant chemotherapy, predominantly (27%, 30/112) with Gemcitabine/Cisplatin regimens and 11 (10%) patients underwent adjuvant radiotherapy (Table 1, Supplementary Table 3). During the follow-up period, 64 (57%) patients recurred and 74 (66%) died. Median follow-up was 25 months, with a median DFS of 12 months and median OS of 25 months. Blood transfusions, microvascular and lymphovascular invasion, lymph node positivity, UICC stage III/IV and adjuvant treatment were significantly associated with DFS and OS, while, preoperative alkaline phosphatase > 100 U/l, preoperative Hemoglobin < 12 g/dl, preoperative C-reactive protein > 10 g/dl, resection status Rx or R1, Comprehensive complication index (CCI) > 40, prolonged hospitalization > 14 days, T category T3 or T4 and intensive care unit stay > 1 day were associated with inferior OS but not DFS. There were no further associations between clinical, demographic or histopathological characteristics and DFS or OS (Table 1).

### DFS and OS in CCA associated with IL-1B C+3954 T SNP

**Figure 1:**
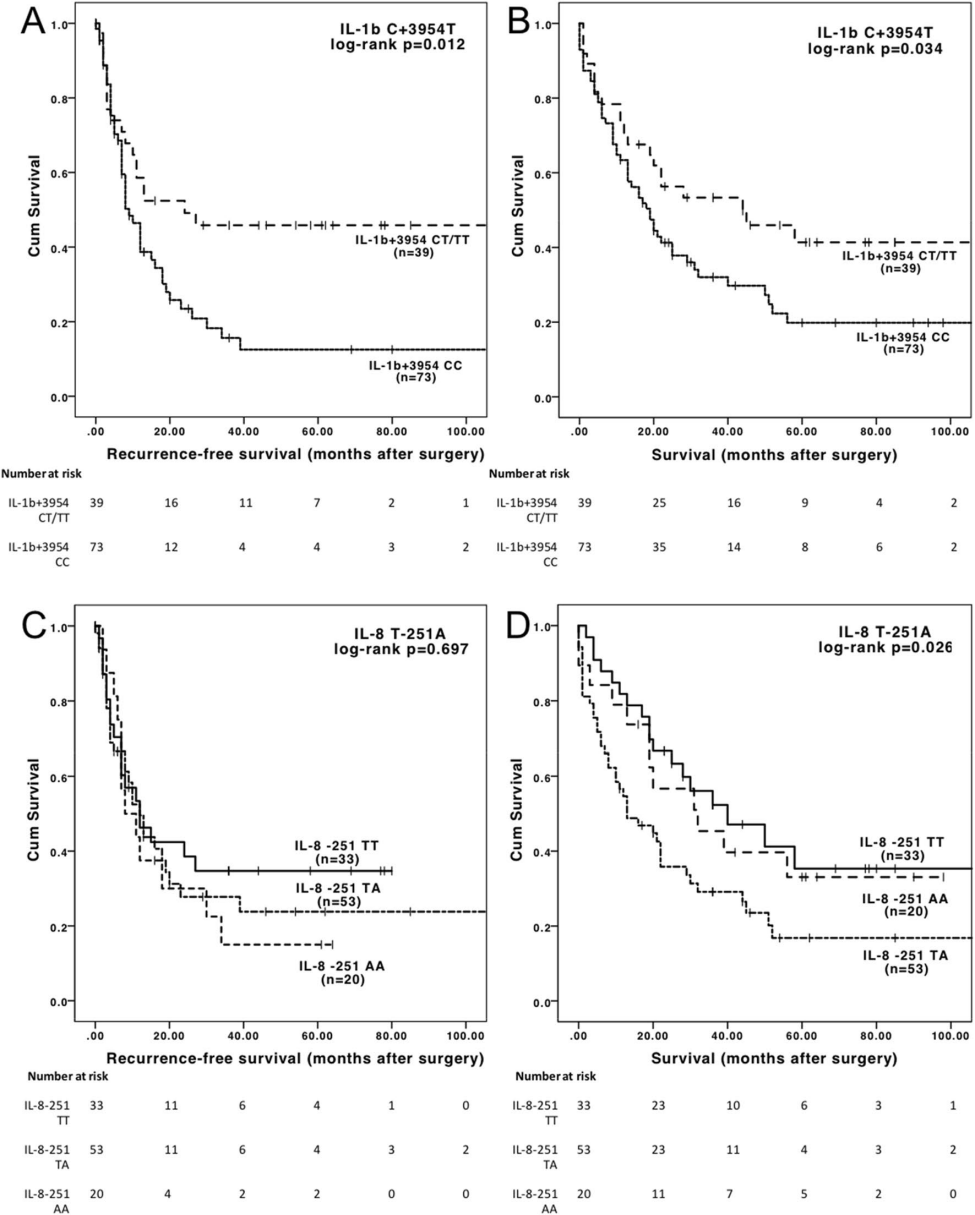
(**A**) Recurrence-free and (**B**) overall survival of patients by *IL-1B* C+3954 T polymorphism. (**C**) Recurrence-free and (**D**) overall survival of patients by *IL-8* T-251A polymorphism. Indicated p values are pooled, the post-hoc pairwise comparisons are as follows for (**D**) *IL-8*-251 TT versus *IL-8*-251 TA log-rank p = 0.013; *IL-8*-251 TT versus *IL-8*-251 AA log-rank *p* = 0.677; *IL-8*-251 TA versus *IL-8*-251 AA log-rank *p* = 0.119.

Genotyping for *IL-1B* C+3954 T (rs1143634) was successful in 112/112 patients (100%), with 65% (73/112) homozygous for the C-allele (C/C), 28% (31/112) heterozygous (C/T) and 7% (8/112) homozygous for the T-allele (T/T), corresponding to allele frequencies of C = 0.790 and T = 0.210, and therefore with great similarity to the reference allele frequencies in European populations, which are C = 0.763 and T = 0.237^23^. The C/T and T/T genotypes were pooled in an additive model (“any T allele”) due to the low incidence of the homozygous T/T genotype. Median DFS for patients with the *IL-1B* +3954 C/C genotype was 9 months (95% CI 5.9–12.1 months, HR = 1.983), while for patients with any T allele (C/T or T/T) it was 24 months (estimates not reached for 95% CI, log-rank p = 0.012, Table 2). Patients homozygous for the *IL-1B* +3954 C-allele (C/C) had a median OS of 19 months (95% CI 13.0–19.0 months, HR = 1.735), while patients with a *IL-1B* +3954 C/T or T/T genotype had a median OS of 44 months (95% CI 3.9–84.0 months, log-rank p = 0.034) (Fig. 1). The clinical variables significantly associated with DFS or OS (Table 1) were equally distributed across the C/C and C/T / T/T groups (Supplementary Table 4).

**Table 2:** Genetic polymorphisms in association with disease-free and overall survival.

Gene, SNP		n, (%)	Disease-free survival (DFS)			Overall survival (OS)		
			Median DFS, m (95% *CI*)	Hazard ratio (95% *CI*)	*p*^†^	Median OS, m (95% *CI*)	Hazard ratio (95% *CI*)	*p*^†^
*VEGF* + 936 (C > T, rs3025039)	CC	76 (67.9)	13 (5.3–20.7)	1 (reference)	.157	21 (10.0–32.0)	1 (reference)	.674
	CT	35 (31.3)	9 (4.0–14.0)	1.425 (0.855–2.375)		20 (12.4–27.6)	1.110 (0.678–1.816)	
	TT	1 (0.9)						
*EGF* + 61 (A > G, rs4444903)	AA	48 (42.9)	16 (8.2–23.8)	1 (reference)	.669	21 (5.5–36.5)	1 (reference)	.753
	AG	64 (57.1)	10 (5.5–14.5)	1.111 (0.676–1.826)		22 (13.8–30.2)	1.077 (0.674–1.721)	
	GG	0 (0.0)						
*EGFR*-1562 (G > A, rs2227983)	GG	38 (33.9)	10 (5.8–14.2)	1 (reference)	.847	22 (7.6–36.4)	1 (reference)	.262
	GA	45 (40.2)	16 (8.2–23.8)	1.013 (0.565–1.815)		28 (6.8–49.2)	1.185 (0.645–2.178)	
	AA	27 (24.1)	8 (.9–15.1)	1.190 (0.605–2.343)		13 (7.1–18.9)	0.737 (0.426–1.277)	
*IL-1B* +3954 (C > T, rs1143634)	TT	8 (7.1)	24 (n.a.)	1 (reference)	**.012**	44 (3.9–84.0)	1 (reference)	**.034**
	CT	31 (27.7)						
	CC	73 (65.2)	9 (5.9–12.1)	1.983 (1.139–3.453)		19 (13.0–25.0)	1.735 (1.043–2.886)	
*IL-6*-174 (G > C, rs1800795)	GG	41 (36.6)	12 (0.0–28.6)	1 (reference)	.413	29 (0.0–69.2)	1 (reference)	.237
	GC	53 (47.3)	12 (6.3–17.7)	1.112 (0.644–1.918)		18 (10.3–25.7)	1.537 (0.914–2.587)	
	CC	16 (14.3)	8 (2.9–13.1)	1.590 (0.781–3.239)		22 (13.1–30.9)	1.465 (0.714–3.004)	
*IL-8*-251 (T > A, rs4073)	TT	33 (29.5)	12 (3.3–20.7)	1 (reference)	.679	40 (14.8–65.2)	1 (reference)	**.026**
	TA	53 (47.3)	12 (7.7–16.3)	1.203 (0.674–2.149)		13 (2.7–23.3)	2.001 (1.144–3.498)	
	AA	20 (17.9)	8 (0.2–15.8)	1.339 (0.661–2.714)		32 (6.3–57.7)	1.177 (0.566–2.445)	
*IL-10*-592 (T > G, rs1800872)	TT	5 (4.5)	12 (6.9–17.1)	1 (reference)	.380	19 (11.9–26.1)	1 (reference)	.928
	TG	53 (47.3)						
	GG	54 (48.2)	10 (5.6–14.4)	1.239 (0.758–2.023)		22 (15.9–28.1)	0.979 (0.616–1.557)	
*CXCR1* + 860(Ex2) (C > G, rs2234671)	CC	102 (91.1)	12 (8.6–15.4)	1 (reference)	.638	20 (16.3–23.7)	1 (reference)	.662
	CG	10 (8.9)	7 (1.9–12.1)	0.789 (0.286–2.175)		25 (0.0–82.8)	0.817 (0.353–1.892)	
	GG	0 (0.0)						
*HIF1α*-1772 (C > T, rs11549465)	CC	88 (78.6)	11 (8.0–14.0)	1 (reference)	.461	19 (11.3–26.6)	1 (reference)	.676
	CT	23 (20.5)	18 (8.1–27.9)	0.804 (0.443–1.459)		28 (5.0–51.0)	0.889 (0.507–1.558)	
	TT	0 (0.0)						
*PTGS2 (COX2)* + 8473 (A > G, rs5275)	AA	49 (43.8)	12 (9.2–14.8)	1 (reference)	.669	20 (8.9–31.0)	1 (reference)	.630
	AG	48 (42.9)	12 (6.3–17.7)	0.988 (0.588–1.661)		25 (11.9–38.1)	0.891 (0.547–1.451)	
	GG	12 (10.0)	18 (2.1–33.9)	0.632 (0.283–1.650)		29 (0.0–62.9)	0.694 (0.307–1.567)	
*PTGS2 (COX2)*-765 (G > C, rs20417)	GG	81 (72.3)	11 (6.9–15.1)	1 (reference)	.993	22 (12.9–31.1)	1 (reference)	.360
	GC	25 (22.3)	13 (3.8–22.3)	0.998 (0.578–1.722)		29 (2.6–55.4)	0.783 (0.459–1.334)	
	CC	4 (3.5)						

Based on Cox proportional hazards model, for DFS including: Blood transfusions, microvascular invasion, lymphovascular invasion, lymph node positivity, UICC stage. For OS including: Alkaline phosphatase, hemoglobin, C-reactive protein, blood transfusions, microvascular invasion, lymphovascular invasion, lymph node positivity, UICC stage, comprehensive complication index and hospitalization. Also see Supplementary Table 2 and 3.

*n.a.* estimates not reached, *CI* confidence interval, *CXCR* chemokine receptor, *EGF* epidermal growth factor, *EGFR* epidermal growth factor receptor, *HIF-1α* hypoxia-inducing factor alpha, *IL* interleukin, *PTGS* prostaglandin-endoperoxide synthase 2, *SNP* single-nucleotide polymorphism, *VEGF* vascular endothelial growth factor, *iCCA* intrahepatic cholangiocarcinoma.

^†^Based on log-rank test.

^$^Significances given for the T/A *vs.* T/T groups and for the “2 unfavorable” *vs.* “0 unfavorable*”* groups.

Significant values are in bold.

### OS in CCA associated with IL-8 T-251A SNP

The genotyping for *IL-8* T-251A (rs4073) was successful in 95% (106/112) of cases, in the remaining 6 cases the quantity of the extracted genomic DNA was insufficient for analysis. Eighteen percent (18%, 20/112) of patients were homozygous for the *IL-8* -251 A-allele (A/A), 47% (53/112) heterozygous *IL-8* -251 T/A and 30% (33/112) homozygous for the T-allele (T/T). Thus, the allele frequencies in our cohort (A = 0.439, T = 0.561) were consistent with the allele frequencies reported in European reference populations (A = 0.454, T = 0.546)^24^. Clinico-pathological characteristics were equally distributed across genotypes (Supplementary Table 5). While *IL-8* T-251A were not significant for DFS, a significant association with survival was observed: Patients with a A/A genotype had a median OS of 32 months (95% CI 6.3–57.7 months), patients with an *IL-8* -251 T/A genotype had a median OS of 13 months (95% CI 2.7–23.3 months, HR 2.001), whereas patients homozygous for the T-allele (T/T) had a median OS of 40 months (95% CI 14.8–65.2 months, HR 1.177, log-rank *p* = 0.026) (Fig. 1).

### Multivariable analysis and combined subgroup analysis

We did not observe statistically significant associations between other tested genes involved in the tumor immune environment and DFS or OS (Table 2). Multivariable analysis of the significant SNPs adjusted for the significant clinico-pathological variables from univariable outcome analysis was performed. DFS did not independently correlate with any SNP. However, multivariable analysis confirmed an independent prognostic effect of the *IL-1B* +3954 (p = 0.013) and the *IL-8* -251 (p = 0.026) polymorphism for OS (Table 3, Supplementary Table 6).

**Table 3:** Multivariable Cox regression analysis of *IL-1B* and *IL-8* polymorphisms disease-free and overall survival in iCCA.

	Disease-free survival (DFS)		Overall survival (OS)	
	Hazard ratio (95% *CI*)^§^	*p*	Hazard ratio (95% *CI*)^#^	*p*
*IL-1B* +3954 C > T *(rs1143634)*		.526		**.013**
C/T or T/T (favorable)	1		1	
C/C (unfavorable)	1.233 (.645–2.360)		2.444 (1.204–4.962)	
*IL-8 -*251 T > A *(rs4073)*		n.a.^&^		**.026**
T/T (favorable)	n.a.^&^		1	
T/A (unfavorable)	n.a.^&^		2.318 (1.158–4.640)	
A/A (unfavorable)	n.a.^&^		1.967 (.363–2.577)	
Combined		n.a.^&^		
0 unfavorable	n.a.^&^		1	**.007**
1 unfavorable	n.a.^&^		0.880 (0.268–2.895)	
2 unfavorable	n.a.^&^		2.395 (0.747–7.895)	

Based on Cox proportional hazards model, for DFS including: blood transfusions, microvascular invasion, lymphovascular invasion, lymph node positivity, UICC stage. For OS including: alkaline phosphatase, hemoglobin, C-reactive protein, blood transfusions, microvascular invasion, lymphovascular invasion, lymph node positivity, UICC stage, comprehensive complication index and hospitalization. Also see Supplementary Table 2 and 3.

*CI* confidence interval, *iCCA* intrahepatic cholangiocarcinoma, *IL* interleukin.

^§^83 patients with complete data were included in the model.

^#^84 patients with complete data were included in the model.

^&^Not significant in univariable analysis (log-rank test), therefore variable was not included in multivariable analysis.

Significant values are in bold.

**Figure 2:**
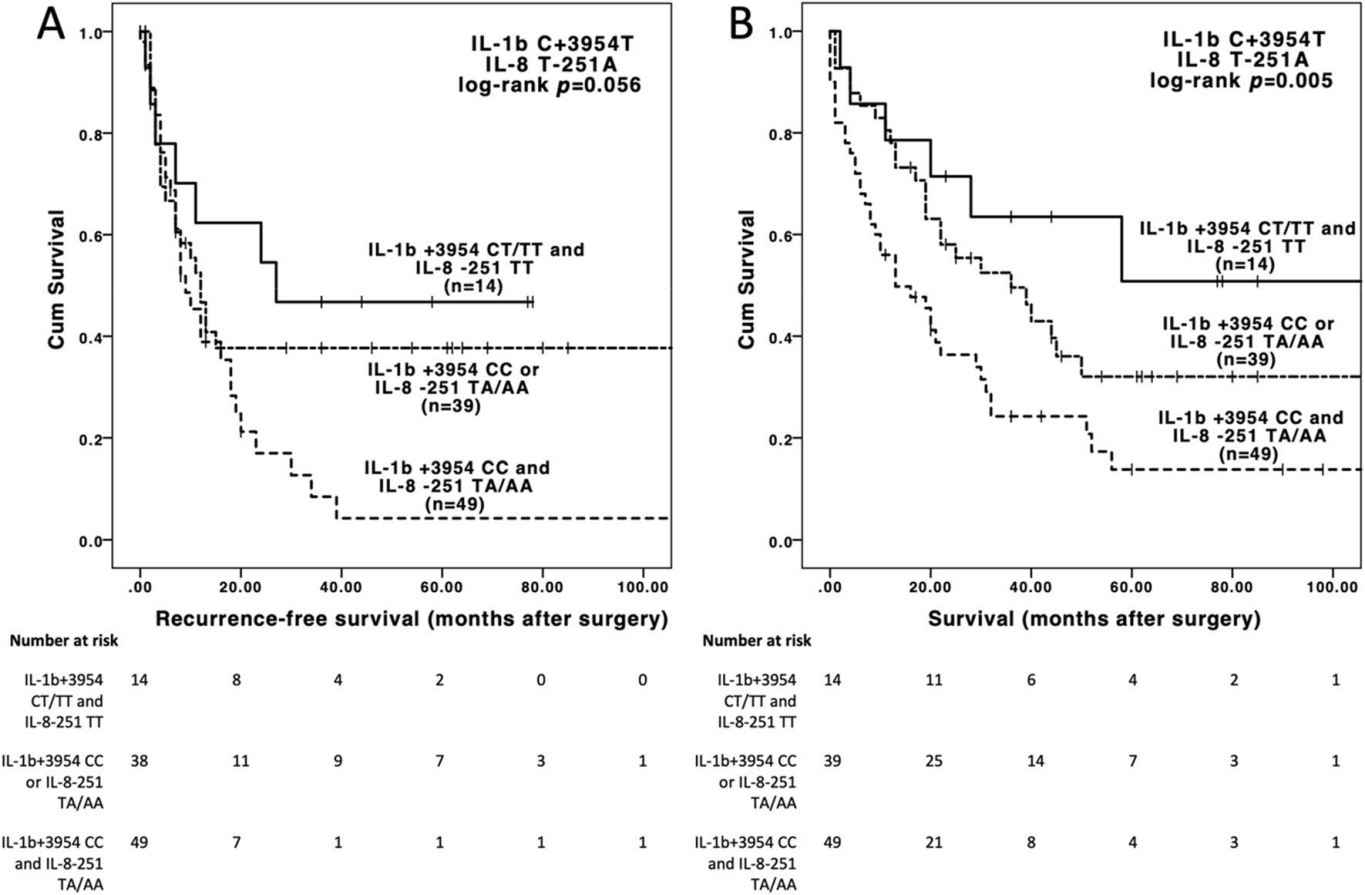
(**A**) Recurrence-free and (**B**) overall survival of patients by 0, 1 or 2 unfavorable *IL-1B* and *IL-8* alleles, *IL-1B* +3954 CC and *IL-8*-251 TA/AA genotypes were considered unfavorable. Indicated p values are pooled, the post hoc pairwise comparisons are as follows: (a) 0 unvafourable versus 1 unfavorable allele log-rank *p* = 0.517; 0 unvafourable versus 2 unfavorable alleles log-rank p = 0.013; 1 unvafourable versus 2 unfavorable alleles log-rank *p* = 0.083. (b) 0 unvafourable versus 1 unfavorable allele log-rank *p* = 0.263; 0 unvafourable versus 2 unfavorable alleles log-rank *p* = 0.01; 1 unvafourable versus 2 unfavorable alleles log-rank *p* = 0.12.

Aiming to establish a novel genetic risk-score based on *IL-1β* +3954 and *IL-8* -251, we further stratified the cohort into patients without unfavorable alleles (*IL-1B* +3954 T/T or T/C genotype and *IL-8* -251 T/T genotype, n = 14), with 1 unfavorable allele (*IL-1B* +3954 C/C genotype or *IL-8* -251 T/A or A/A genotype, n = 39) and with 2 unfavorable alleles (*IL-Bβ* +3954 C/C genotype and *IL-8* -251 T/A or A/A genotype, n = 49). While this stratification did not reach a significant association with DFS (p = 0.056, Fig. 2A), it was significantly associated with OS (no unfavorable allele, 36 months median OS, 1 unfavorable allele, 25 months median OS, 2 unfavorable alleles, 13 months median OS, p = 0.005, Fig. 2B). Multivariable analysis with significant clinico-pathological characteristics from univariable analysis confirmed the independent prognostic effect of this allele grouping (p = 0.007, Table 3, Supplementary Table 7).

## Conclusion

Intrahepatic CCA is a relatively rare, but highly aggressive gastrointestinal malignancy that frequently recurs even after major liver resections^7^. In this study, we analyzed polymorphisms in genes driving tumor-associated immunosuppression and neovascularization to determine their prognostic value in a large and homogenous Western cohort of iCCA patients. As such, we found that patients with the *IL-1B* +3954 C/C genotype had shorter DFS and OS, while patients with an *IL-8* -251 T/A or A/A genotype had shorter OS. Both polymorphisms were confirmed as independent prognostic factors for OS in multivariable analysis. Combining these allowed for patient stratification into survival groups by the number of unfavorable alleles.

IL-1β signals through binding to the receptor IL-1R1, which is widely expressed on various leucocyte populations and frequently across epithelial tissues^25^. Physiological effects include the expansion of hematopoietic progenitors, regulation of emergency hematopoiesis and prolonged survival of neutrophils and monocytes-macrophages^26^. The oncological relevance of IL-1β signaling was recently demonstrated in IL1β-deficient mice, which showed inhibited tumor growth in various tumor entities and retained antitumor immunity^27^. IL-1β signaling drives carcinogenesis by several mechanisms, including sustained inflammation with preferential macrophage and neutrophil recruitment, angiogenesis and immunosuppression^28^.

Recently, a TME-based prognostic classification of iCCA identified a distinct M2-polarized macrophage-dominated subtype (I3), which was associated with inferior survival compared to subtypes devoid of any immune infiltration (I1) and lymphoid- and myeloid-enriched tumors (I2)^29^. Interestingly, the potential of targeting IL-1β-mediated cancer immune evasion has been translated into clinical trials in other gastrointestinal malignancies^30^.

In our cohort, patients with the *IL-1B* +3954 (rs1143634) had a median OS of 19 months as opposed to 44 months with a C/T or T/T genotype. Functional data on the *IL-1B* rs1143634 SNP is limited to non-oncological studies, with evidence that in the systemic circulation, the SNP translates to higher IL-1β production by monocytes without any qualitative changes of the protein, both *in vitro*^31^ and at sites of infection^32^. Due to a lack of functional data from hepatic or tumor immunology, the exact effects on the CCA TME remain to be determined.

We furthermore observed an independent association of *IL-8* T-251A SNP with OS. IL-8 signaling has been previously identified as a central regulator of VEGF-independent and HIF1α-independent angiogenesis in gastrointestinal malignancies^33^, signaling through the CXCR1/CXCR2 receptors^34^. Typical origins of IL-8 in the iCCA TME are suggested to be endothelial cells and CAFs, along with infiltrating myeloid cells^35,36^. CXCR1 is physiologically found on granulocytes, monocytes, mast cells and natural killer cells, but also on cancer cells and the TME, where the signaling mediates immunosuppressive responses^34^.

Previously, the *IL-8* -251 T > A polymorphism has been linked to shortened DFS in stage III colon cancer and to shortened DFS and OS in localized gastric cancer^20,37^. The SNP is localized in the *IL-8* promoter region and effects a higher expression of IL-8 with higher serum levels compared to wildtype individuals^38^. Furthermore, the IL-8 -251 A allele has been associated to increased IL-8 mucosal tissue levels, inflammation, metaplasia and carcinogenesis in individuals with Helicobacter pylori infection^39^. In this study, no clear association of the A/A genotype with DFS or OS was demonstrated. Only a small subgroup of our cohort (20 patients, 17.9%) harbored the *IL-8* -251 A/A genotype, potentially increasing the risk of type 2 error, and, at the same time, the risk of type 1 error for significant findings for the TA subgroup.

Our group was the first to recently describe a relevant prognostic value of gene polymorphisms in patients with CCA^40^. As such, the *CXCR1 (rs2234671)* SNP, (IL-8 receptor) was associated with decreased DFS and OS in surgical pCCA patients. This polymorphism is presumed to enhance intracellular CXCR1-signaling, leading to stronger IL-8 effects^41^. In keeping with the prognostic effects of *IL-8* variations observed in this study, this underlines the importance of the IL-8 pathway in the TME of CCA. In keeping with emerging evidence on biological differences of the two tumor localizations^3^, this difference in the relevance of prognostic polymorphisms supports the concept of pluralistic roles in the TME.

As with most clinical outcome studies, this analysis has some inherent limitations. First, this is a retrospective, single-center analysis that requires prospective external validation. Second, while this is a very homogenous cohort in terms of patient selection and surgical approach, the present study completed recruitment in 2019, the same year when the BILCAP study provided universal level I evidence for adjuvant capecitabine treatment^42^. However, sufficiently powered biomarker studies with long-term outcomes in this rare tumor entity may require several more years to complete patient recruitment under the BILCAP selection criteria. Third, while the exclusion of patients with extrahepatic spread and inoperable disease afforded an extremely homogenous patient cohort, our findings may not be representative of all patients with iCCA. Fourth, while this cohort is relatively large for a single-center study, the relatively low total number of events leads to a risk of statistical overfitting, thus warranting further external validation. Fifth, we examined only ten genes in a pathway-driven approach, with the potential to expand the current analysis to larger gene panels. Our preliminary findings should thus be regarded as hypothesis-generating until confirmed in independent cohorts.

The potential of the present study, compared to other prognostic factors for hepatobiliary malignancies, is the fact that genetic variants can be accessed from any genetic material, including blood leucocytes, thus constituting a potential preoperative biomarker. As patients with iCCA often require extensive and high-risk resections, the present study may contribute to preoperative oncological and outcome considerations.

This study in a large and homogenously treated iCCA cohort reveals a potential prognostic value of the *IL-1B* +3954 and the *IL-8* -251 polymorphism for OS after curative-intent surgery for iCCA, consistent with our hypothesis that genetic variants of tumor-mediated immune suppression and angiogenesis may have additional clinical value for prognostic patient stratification. Potentially, our findings may translate into the identification of novel therapeutic targets for this understudied tumor entity. Biomarker-embedded clinical trials and a validation in independent patient cohorts are required to confirm our findings.

## Supplementary Information

Supplementary Information.

## Data Availability

The datasets generated and/or analysed during the current study are available in the ClinVar repository (ClinVar assession number: SCV004011734—SCV004011744, summary report: https://submit.ncbi.nlm.nih.gov/api/2.0/files/9wosnzv0/sub13574875__100__submitter_report_b.txt/?format=attachment). Further relevant data were reported within the article. Further supporting data will be provided upon written request addressed to the corresponding author.
