# Supplementary material for: Genetic polymorphisms in interleukin-1β (rs1143634) and interleukin-8 (rs4073) are associated with survival after resection of intrahepatic cholangiocarcinoma
Source: Sci Rep. 2023 Jul 28;13:12283. doi: 10.1038/s41598-023-39487-7 (PMC10382511; doi:10.1038/s41598-023-39487-7)
Supplement: Supplementary file 1 — Supplementary Information. [file 41598_2023_39487_MOESM1_ESM.docx]

***Supplementary Material***

**Genetic polymorphisms in interleukin-1β (rs1143634) and**

**interleukin-8 (rs4073) predict survival in patients with intrahepatic**

**cholangiocarcinoma**

Isabella Lurje, Nadine Therese Gaisa, Edgar Dahl, Ruth Knüchel, Pavel Strnad, Christian Trautwein, Frank Tacke, Ulf Peter Neumann, Zoltan Czigany, Georg Lurje

**Table of contents**

**Supplementary Figure 1,** CONSORT flow diagram of the study design and patient incusion/ exclusion criteria

**Supplementary Table 1,** Gene and SNP selection and their role in the cholangiocarcinoma microenvironment (p. 2)

**Supplementary Table 2**, Primer sequences, restriction enzymes and reaction conditions (p. 3)

**Supplementary Table 3**, Patient Characteristics (pp. 4-6)

**Supplementary Table 4,** Selected clinico-pathological characteristics and their distribution across the *IL-1B* +3954 genetic variants

**Supplementary Table 5,** Selected clinico-pathological characteristics and their distribution across the *IL-8* -251 genetic variants

**Supplementary Table 6,** Full multivariable analysis of clinico-pathological factors and Disease-Free and Overall Survival for the *IL-1B*+3954 and the *IL-8-*251 polymorphism, individually (p. 7)

**Supplementary Table 7,** Full multivariable analysis of clinico-pathological factors and Disease-Free and Overall Survival for the *IL-1B*+3954 and the *IL-8*-251 polymorphism, in a combined model by number of unfavorable alleles

**Additional References** (pp. 12-13)

**Supplementary Figure 1**: CONSORT flow diagram of the study design and patient incusion/ exclusion criteria.


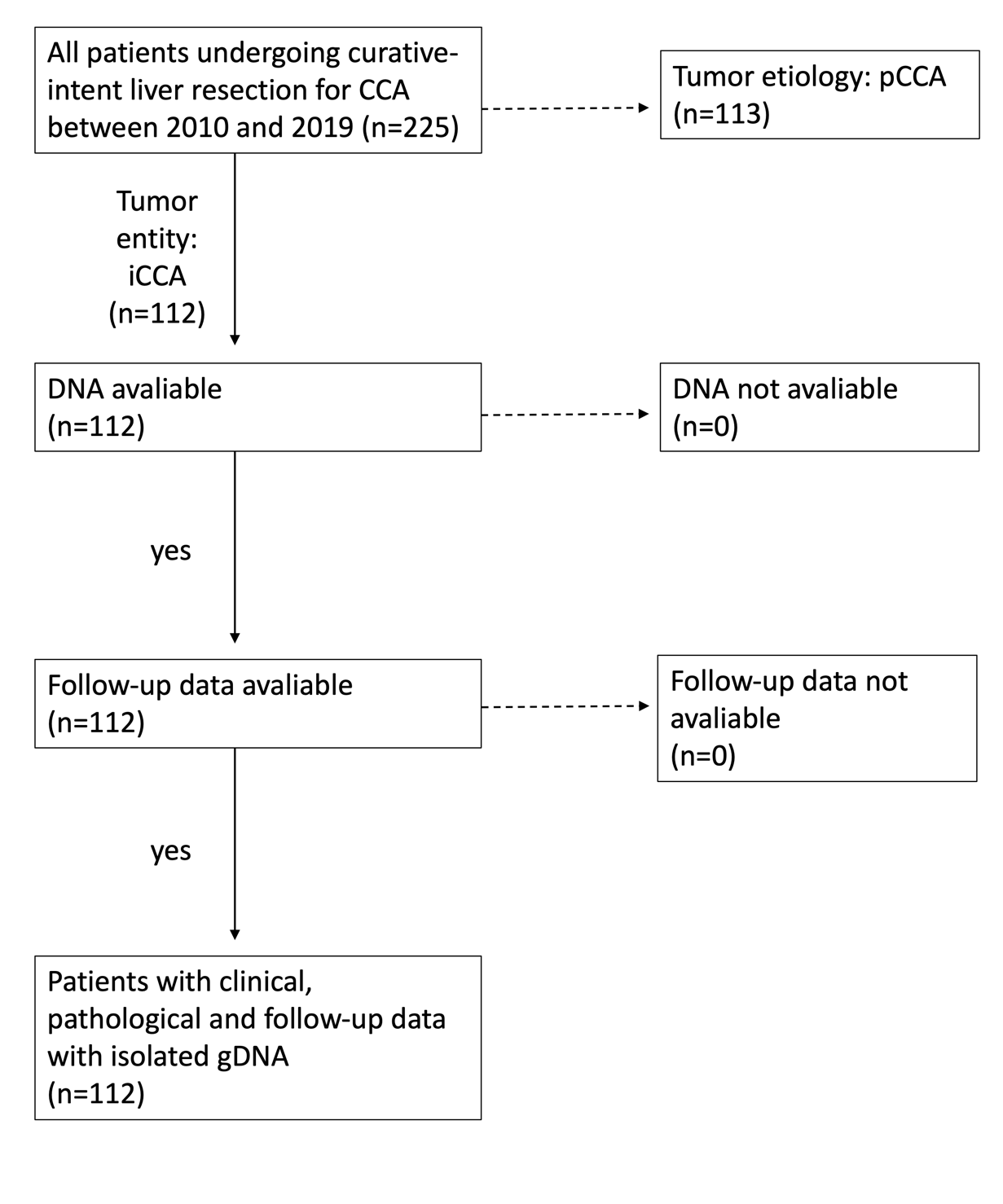


Abbreviations: CCA, cholangiocarcinoma; dDNA, genomic DNA; iCCA, intrahepatic cholangiocarcinoma; pCCA, perihilar Cholangiocarcinoma.

**Supplementary Table 1: Gene and SNP selection and their role in the cholangiocarcinoma microenvironment**

| Gene (Allele, SNP) | Selected SNP effects | Gene/ Protein Function in cancer |
| --- | --- | --- |
| *VEGF*+936  (C>T, rs3025039) | 936T allele: lower VEGF plasma levels [41], shorter DFS in stage III colon cancer [11] | Stimulates angiogenesis and attracts circulating monocytes [10], VEGF signaling implicated in poor survival of CCA patients [42] |
| *EGF*+61  (A>G, rs4444903) | 61G allele: higher susceptibility to gallbladder cancer [43], higher EGF production from PBMCs [44] | EGF/EGFR axis contributes to EMT in CCA [45] |
| *EGFR*-1562  (G>A, rs2227983) | -1562A allele: Missense variant, unfavourable survival in esophageal SCC patients [46] | EGF/EGFR axis contributes to EMT in CCA [45], EGFR overexpression associated with decreased OS in iCCA [47] |
| *IL-1B*+3954  (C>T, rs1143634) | 3954T allele: shorter DFS in stage II colon cancer [20] | Tumor- and TME-derived IL-1β promotes progression and metastasis through recruitment of T-cell-suppressive neutrophils [14] |
| *IL-6*-174  (G>C, rs1800795) | 174C allele: lower IL-6 plasma levels [48] | IL-6 signature is activated in vascular CAFs and drives tumor progression of iCCA [9] |
| *IL-8*-251  (T>A, rs4073) | 251A allele: shorter DFS in stage III colon cancer [49] | VEGF-independent tumor angiogenesis, IL-8 protein expression in pCCA associated with increased microvessel density and decreased OS [50] |
| *IL-10*-592  (T>G, rs1800872) | 592G allele: higher IL-10 plasma levels [51] | Immunosuppressive effects on DCs and subsequent T cell priming and cytotoxicity against CCA [52] |
| *CXCR1*+860(Ex2)  (C>G, rs2234671) | 860G allele: inferior DFS and OS in pCCA, alteration of tertiary protein structure of the receptor [53] | IL-8 receptor on granulocytes, monocytes, mast cells [34],  CXCR1 is often overexpressed in the TME, with proangiogenic and immunosuppressive effects [54] |
| *HIF-1α*-1772  (C>T, rs11549465) | 1772T allele: Higher *HIF-1α* transcription activity under norm- and hypoxia [55] | HIF-1α mediates tumor immune suppression, including upregulation of PD-L1 on antigen-presenting cells [56] and the expansion of TAMs [57] |
| *PTGS2*  *(COX-2)*+8473  (A>G, rs5275) | G allele: lower promoter activity, lower gene expression [58] | COX-2 directly enhances growth of CCA cells, is expressed by tumor cells [59]  Immunosuppressive effects: inhibition of CTL function [60] |
| *PTGS2*  *(COX-2)*-765  (G>C, rs20417) | C allele: lower promoter activity [61] |  |

Abbreviations: CAF, Cancer-associated fibroblast; CCA, cholangiocarcinoma; COX, cyclooxygenase; CTL, cytotoxic T lymphocyte; CXCR, chemokine receptor; DC, dendritic cell; DFS, disease-free survival; EGF, epidermal growth factor; EGFR, epidermal growth factor receptor; EMT, epithelial-to-mesenchymal transition; HCC, hepatocellular carcinoma; HIF-1α, hypoxia-inducing factor alpha; iCCA, intrahepatic cholangiocarcinoma; IL, interleukin; NK, natural killer; OS, overall survival; PBMC, Peripheral Blood Mononuclear Cell; pCCA, perihilar cholangiocarcinoma; PD-L1, programmed death-ligand 1; PTGS, Prostaglandin-Endoperoxide Synthase; SCC, squamous cell carcinoma; SNP – single nucleotide polymorphism; TAM, tumor-associated macrophages; TME, tumor microenvironment; VEGF, vascular endothelial growth factor.

**Supplementary Table 2: Primer sequences, restriction enzymes and reaction conditions**

| **Gene (Allele, SNP)** | **Forward Primer Sequence** | **Reverse Primer Sequence** | **Annealing Tempera-ture (°C)** | **Restriction Enzyme** |
| --- | --- | --- | --- | --- |
| ***VEGF*+936**  (C>T, rs3025039) | AGA CTC CGG CGG AAG CAT | TGT ATG TGG GTG GGT GTG TC | 60 | NIaIII |
| ***EGF*+61**  (A>G, rs4444903) | CAT TTG CAA ACA GAG GCT CA | TGT GAC AGA GCA AGG CAA AG | 60 | AluI |
| ***EGFR*-1562**  (G>A, rs2227983) | TGC TGT GAC CCA CTC TGT CT | CCA GAA GGT TGC ACT TGT CC | 59 | BstNI |
| ***IL-1B*+3954**  (C>T, rs1143634) | GTT GTC ATC CAG ACT TTG ACC | TTC AGT TCA TAT GGA CCA GA | 58 | TaqI |
| ***IL-6*-174**  (G>C, rs1800795) | GCC TCA ATG ACG ACC TAA GC | TCA TGG GAA AAT CCC ACA TT | 55 | NIaIII |
| ***IL-8*-251**  (T>A, rs4073) | TGC CAT TAA AAG AAA ATC ATC CA | CAT TTA AAA TAC TGA AGC TCC ACA | 56 | MfeI |
| ***IL-10*-592**  (T>G, rs1800872) | GAG CAC TAC CTG ACT AGC ATA TAA G | GTG GGC TAA ATA TCC TCA AAG T | 60 | RsaI |
| ***Cxcr1*+860** (Ex2)  (C>G, rs2234671) | CTC ATG AGG ACC CAG GTG AT | GGT TGA GGC AGC TAT GGA GA | 60 | AluI |
| ***HIF-1a*-1772**  (C>T, rs11549465) | CCC AAT GGA TGA TGA CTT CC | AGT GGT GGC ATT AGC AGT AGG | 59 | Tsp45I |
| ***PTGS2 (COX-2)*** +8473  (A>G, rs5275) | GTT TGA AAT TTT AAA GTA CTT TTG AT | TTT CAA ATT ATT GTT TCA TTG C | 53 | BclI |
| ***PTGS2 (COX-2)***-765  (G>C, rs20417) | ATT CTG GCC ATC GCC GCT TC | CTC CTT GTT TCT TGG AAA GAG ACG | 55 | AciI |

Abbreviations: COX, cyclooxygenase; CXCR, chemokine receptor; EGF, epidermal growth factor; EGFR, epidermal growth factor receptor; HIF-1α, hypoxia-inducing factor alpha; IL, interleukin; PTGS, prostaglandin-endoperoxide synthase 2; SNP, single-nucleotide polymorphism; VEGF, vascular endothelial growth factor.

**Supplementary Table 3: Patient Characteristics**

| **Patient Characteristics** | **iCCA (n=112)** |
| --- | --- |
| **age (years)** | 65 ± 11.5 |
| **BMI** | 26 ± 4.8 |
| **sex ratio (F:M),** n,(%) | 64 (57.1) : 48 (42.9) |
| **ASA,** n(%) |  |
| I | 4 (3.6) |
| II | 46 (41.1) |
| III | 56 (50.0) |
| IV | 5 (4.5) |
| preoperative cholangitis, n,(%) | 10 (8.9) |
| **EBD** (Stent), n (%) | 17 (15.2) |
| unilateral | 14 (12.5) |
| bilateral | 3 (2.7) |
| **PBD**, n (%) | 1 (0.9) |
| unilateral | 0 |
| bilateral | 1 (0.9) |
| Portal vein embolization, n,(%) | 11 (9.8) |
| Neoadjuvant chemotherapy, n,(%) | 3 (2.7) |
| Laparoscopic approach n,(%) | 9 (8.0) |
| **Operative procedure** n,(%) |  |
| Atypical/anatomical resection/ bisegmentectomy | 26 (23.0) |
| Right hepatectomy | 19 (17.0) |
| Left hepatectomy | 16 (14.3) |
| Extended right hepatectomy | 14 (12.5) |
| Extended left hepatectomy | 9 (8.0) |
| Right trisectorectomy | 6 (5.4) |
| Left trisectorectomy | 9 (8.0) |
| Hepatoduodenectomy | 0 (0.0) |
| ALPSS | 12 (10.7) |
| Lymphadenectomy | 95 (84.8) |
| Vessel replacement n,(%) | 64 (57.1) |
| venous | 64 (57.1) |
| arterial | 0 (0.0) |
| both | 0 (0.0) |
| Knife-to-skin time (min) | 303 ± 113.7 |
| Intraoperative blood transfusions (U) | 1.2 ± 3.1 |
| Intraoperative FFP (U) | 2.3 ± 4.6 |
| T category, n (%) |  |
| Tis | 1 (0.9) |
| T1 | 38 (34.0) |
| T1a | 19 (17.0) |
| T1b | 19 (17.0) |
| T2 | 62 (55.4) |
| T3 | 5 (4.5) |
| T4 | 4 (3.6) |
| N category, n (%) |  |
| N0 | 59 (52.7) |
| N1 | 39 (34.8) |
| R category, n (%) |  |
| R0 | 84 (75.0) |
| R1 | 15 (13.4) |
| Rx | 9 (8.0) |
| (Micro-)vascular invasion, n (%) | 39 (34.8) |
| Portal vein infiltration, n (%) | 4 (3.6) |
| Hepatic artery infiltration, n (%) | 0 (0.0) |
| Lymphovascular invasion, n (%) | 28 (25.0) |
| Perineural invasion, n (%) | 25 (22.3) |
| Tumor grading, n (%) |  |
| G1 | 1 (0.9) |
| G2 | 64 (57.1) |
| G2-3 | 5 (4.5) |
| G3 | 29 (25.9) |
| G4 | 3 (2.7) |
| Tumor stage, UICC (8^th^ ed), n (%) |  |
| 0 | 2 (1.8) |
| I | 25 (22.3) |
| Ia | 14 (12.5) |
| Ib | 11 (9.8) |
| II | 29 (25.9) |
| III | 37 (33.0) |
| IIIa | 2 (1.8) |
| IIIb | 36 (32.1) |
| IIIc |  |
| IV | 5 (4.5) |
| Cumulative ICU stay, days | 3.7 ± 9.4 |
| Hospitalization, days | 18.1 ± 14.3 |
| **Postoperative complications**, n (%) |  |
| No complications | 31 (27.7) |
| Clavien-Dindo I | 3 (2.7) |
| Clavien-Dindo II | 30 (26.8) |
| Clavien-Dindo IIIa | 20 (17.9) |
| Clavien-Dindo IIIb | 9 (8.0) |
| Clavien-Dindo IVa | 10 (8.9) |
| Clavien-Dindo IVb | 0 (0.0) |
| Clavien-Dindo V | 9 (8.0) |
| Calculated CCI | 32.8 ± 30.5 |
| Adjuvant Radiotherapy | 11 (9.8) |
| Adjuvant Chemotherapy | 57 (50.9) |
| Gemcitabine | 3 (2.7) |
| Gemcitabine+ Cisplatin | 30 (26.8) |
| Other | 24 (21.1) |

Data presented as mean and standard deviation if not noted otherwise.

Abbreviations: ALPPS, Associating Liver Partition with Portal Vein Ligation for Staged Hepatectomy; ASA, American society of anesthesiologists classification; BMI, body mass index; CCI, comprehensive complication index; EBD, endoscopic biliary drainage; FFP, fresh frozen plasma; iCCA, intrahepatic cholangiocarcinoma; ICU, intensive care unit; PBD, percutaneous biliary drainage; UICC, Union internationale contre le cancer.

Pathological categories are based on TNM 8, UICC Stage 8 versions.

Patients were classified as having received chemotherapy or radiotherapy if they received at least one cycle of the respective adjuvant treatment.

**Supplementary Table 4: Selected clinico-pathological characteristics and their distribution across the *IL-1B* +3954 genetic variants**

|  | *IL-1B*+3954 CC | *IL-1β*+3954 CT/TT | *p*=^&^ |
| --- | --- | --- | --- |
| Alkaline phosphatase, >100 U/l | 49 (68.1) | 24 (63.2) | .605 |
| Hemoglobin ≤ 12 g/l | 20 (27.8) | 9 (23.7) | .643 |
| C-reactive protein > 10 mg/l | 39 (54.2) | 14 (36.8) | .084 |
| Blood transfusions | 26 (35.6) | 11 (28.2) | .427 |
| Microvascular invasion | 29 (41.4) | 10 (29.4) | .235 |
| Lymphovascular invasion | 20 (29.4) | 8 (23.5) | .530 |
| Lymph node positivity | 30 (44.8) | 11 (34.4) | .326 |
| UICC stage III-IV | 32 (48.5) | 11 (33.3) | .152 |
| Comprehensive complication index (CCI) > 40 | 27 (37.5) | 11 (28.2) | .324 |
| Hospitalization > 14 days | 37 (50.7) | 13 (33.3) | .078 |
| Adjuvant chemotherapy | 40 (57.1) | 17 (43.6) | .174 |
| Adjuvant radiotherapy | 8 (11.8) | 3 (7.7) | .504 |

**Parameters significantly associated with DFS or OS on univariable analysis are tested for their distribution across *IL-1B* +3954 genotypes.**

Numbers given as n, % if not indicated otherwise

^&^ based on Pearson Chi-Square test,

**Supplementary Table 5: Selected clinico-pathological characteristics and their distribution across the *IL-8* -251 genetic variants**

|  | *IL-8* -251 AA | *IL-8* -251 TA | *IL-8* -251 TT | *p*=^%^ |
| --- | --- | --- | --- | --- |
| Alkaline phosphatase, >100 U/l | 16 (80.0) | 33 (62.3) | 20 (62.5) | .327 |
| Hemoglobin ≤ 12 g/l | 5 (25.0) | 14 (26.4) | 10 (31.3) | .853 |
| C-reactive protein > 10 mg/l | 8 (40.0) | 24 (45.3) | 17 (53.1) | .627 |
| Blood transfusions | 6 (30.0) | 18 (34.0) | 12 (36.4) | .894 |
| Microvascular invasion | 4 (22.2) | 23 (45.1) | 11 (36.7) | .223 |
| Lymphovascular invasion | 1 (5.9) | 18 (35.3) | 8 (26.7) | .063 |
| Lymph node positivity | 6 (33.3) | 23 (48.9) | 10 (34.5) | .341 |
| UICC stage III-IV | 8 (40.0) | 22 (46.8) | 11 (40.7) | .822 |
| Comprehensive complication index (CCI) > 40 | 3 (15.0) | 23 (43.4) | 9 (28.1) | .054 |
| Hospitalization > 14 days | 6 (30.0) | 28 (52.8) | 13 (39.4) | 170 |
| Adjuvant chemotherapy | 8 (42.1) | 28 (52.8) | 14 (42.4) | .558 |
| Adjuvant radiotherapy | 2 (10.5) | 4 (7.7) | 4 (12.7) | .763 |

**Parameters significantly associated with DFS or OS on univariable analysis are tested for their distribution across *IL-8 -251* genotypes.**

Numbers given as n, % if not indicated otherwise

^%^ based on Pearson Chi-Square test,

**Supplementary Table 6: Full multivariable analysis of clinico-pathological factors and Disease-Free and Overall Survival for the *IL-1B*+3954 and the *IL-8*-251 polymorphism, individually**

|  | **Disease-free survival** | | **Overall survival** | |
| --- | --- | --- | --- | --- |
|  | HR (95%CI)^§^ | *p*= | HR (95%CI)^#^ | *p*= |
| *IL-1B*+3954 C/C | 1.233 (.645-2.360) | .526 | 2.444 (1.204-4.962) | **.013** |
| *IL-8-*251*T/A* | n.a.^&^ |  | 2.318 (1.158-4.640) | **.026** |
| *-*251*A/A* |  |  | 1.967 (.363-2.577) |  |
| AP >100 U/l | n.a.^&^ |  | 1.086 (.501-2.352) | .930 |
| Hemoglobin, <12 g/dl | n.a.^&^ |  | 1.736 ( .818-3.686) | **.**138 |
| CRP >10 g/dl | n.a.^&^ |  | 1.049 (.519-2.122) | .429 |
| Blood transfusions | 1.696 (.920-3.127) | .090 | 1.170 (.589-2.325) | .725 |
| Rx or R1 status | n.a.^&^ |  | 1.957 (.973-3.938) | .245 |
| Microvascular invasion | 1.473 (.739-2.934) | .271 | 3.559 (1.606-7.887) | **.002** |
| Lymphovascular invasion | 1.042 (.384-2.829) | .936 | 5.705 (2.139-15.217) | **.001** |
| Lymph node positivity | 1.155 (.305-4.376) | .833 | 1.964 (.419-9.209) | .392 |
| UICC stage III/IV | 2.904 (.818-10.316) | **.**099 | 3.716 (.878-15.720) | .074 |
| CCI>40 | n.a.^&^ |  | 1.100 (.455-2.657) | .832 |
| Hospitalization >14 days | n.a.^&^ |  | 1.375 (.516-3.664) | .524 |

^§^83 patients with complete data were included in the model

^#^84 patients with complete data were included in the model

^&^not significant in univariable analysis (log-rank test)

Abbreviations: AP, alkaline phosphatase; CCI, comprehensive complication index; ICU, intensive care unit; R, resection margin status; UICC, union internationale contre le cancer.

Due to multicollinearity, the following variables were not included in the multivariable analysis: adjuvant treatment (in the pre-BILCAP study era, patient selection for adjuvant treatment was based on resection margin and L and N status), T category (factors into the UICC category), ICU stay (collinearity with CCI and hospitalization).

**Supplementary Table 7: Full multivariable analysis of clinico-pathological factors and Disease-Free and Overall Survival for the *IL-1B*+3954 and the *IL-8*-251 polymorphism, in a combined model by number of unfavorable alleles**

|  | **Disease-free survival** | | **Overall survival** | |
| --- | --- | --- | --- | --- |
|  | HR (95%CI)^§^ | *p*= | HR (95%CI)^#^ | *p*= |
| Combined alleles |  |  |  | **.007**^$^ |
| 1 unfavorable | 0.418 (0.156-1.120) | .197 | 0.880 (.268-2.895) |  |
| 2 unfavorable | 0.589 (0.224-1.549) |  | 2.395 (.747-7.895) |  |
| AP >100 U/l | n.a.^&^ |  | 1.265 (.584-2.741) | .551 |
| Hemoglobin, <12 g/dl | n.a.^&^ |  | 1.544 (.737-3.232) | **.**249 |
| CRP >10 g/dl | n.a.^&^ |  | 1.025 (.502-2.097) | .945 |
| Blood transfusions | 1.613 (.843-3.089) | .149 | .965 (.472-1.974) | .923 |
| Rx or R1 status | n.a.^&^ |  | 2.261 (1.088-4.699) | **.029** |
| Microvascular invasion | 0.618 (.298-1.279) | .195 | 3.297 (1.496-7.267) | **.003** |
| Lymphovascular invasion | 1.012 (.372-2.751) | .936 | 5.705 (2.139-15.217) | **.000** |
| Lymph node positivity | 1.259 (.333-4.764) | .734 | 2.410 (.544-10.672) | .247 |
| UICC stage III/IV | 3.688 (1.035-13.137) | **.044** | 4.526 (1.134-18.057) | **.032** |
| CCI>40 | n.a.^&^ |  | 1.030 (.413-2.565) | .950 |
| Hospitalization >14 days | n.a.^&^ |  | 1.589 (.600-4.212) | .351 |

^§^83 patients with complete data were included in the model

^#^84 patients with complete data were included in the model

^&^not significant in univariable analysis (log-rank test)

^$^ significances given for the “2 unfavorable” vs. “0 unfavorable” groups.

Abbreviations: AP, alkaline phosphatase; CCI, comprehensive complication index; CRP, C-reactive protein; ICU, intensive care unit; R, resection margin status; UICC, union internationale contre le cancer.

Due to multicollinearity, the following variables were not included in the multivariable analysis: adjuvant treatment (in the pre-BILCAP study era, patient selection for adjuvant treatment was based on resection margin and L and N status), T category (factors into the UICC category), ICU stay (collinearity with CCI and hospitalization).

**Supplementary References:**

41. Renner W, Kotschan S, Hoffmann C, Obermayer-Pietsch B, Pilger E. A common 936 C/T mutation in the gene for vascular endothelial growth factor is associated with vascular endothelial growth factor plasma levels. J Vasc Res. 2000;37(6):443-8.

42. Andersen JB, Spee B, Blechacz BR, Avital I, Komuta M, Barbour A, et al. Genomic and genetic characterization of cholangiocarcinoma identifies therapeutic targets for tyrosine kinase inhibitors. Gastroenterology. 2012;142(4):1021-31.e15.

43. Vishnoi M, Pandey SN, Modi DR, Kumar A, Mittal B. Genetic susceptibility of epidermal growth factor +61A>G and transforming growth factor beta1 -509C>T gene polymorphisms with gallbladder cancer. Human immunology. 2008;69(6):360-7.

44. Shahbazi M, Pravica V, Nasreen N, Fakhoury H, Fryer AA, Strange RC, et al. Association between functional polymorphism in EGF gene and malignant melanoma. Lancet (London, England). 2002;359(9304):397-401.

45. Clapéron A, Mergey M, Nguyen Ho-Bouldoires TH, Vignjevic D, Wendum D, Chrétien Y, et al. EGF/EGFR axis contributes to the progression of cholangiocarcinoma through the induction of an epithelial-mesenchymal transition. Journal of hepatology. 2014;61(2):325-32.

46. Yang PW, Hsieh MS, Huang YC, Hsieh CY, Chiang TH, Lee JM. Genetic variants of EGF and VEGF predict prognosis of patients with advanced esophageal squamous cell carcinoma. PloS one. 2014;9(6):e100326.

47. Yang X, Wang W, Wang C, Wang L, Yang M, Qi M, et al. Characterization of EGFR family gene aberrations in cholangiocarcinoma. Oncol Rep. 2014;32(2):700-8.

48. Fishman D, Faulds G, Jeffery R, Mohamed-Ali V, Yudkin JS, Humphries S, et al. The effect of novel polymorphisms in the interleukin-6 (IL-6) gene on IL-6 transcription and plasma IL-6 levels, and an association with systemic-onset juvenile chronic arthritis. The Journal of clinical investigation. 1998;102(7):1369-76.

49. Lurje G, Zhang W, Schultheis AM, Yang D, Groshen S, Hendifar AE, et al. Polymorphisms in VEGF and IL-8 predict tumor recurrence in stage III colon cancer. Annals of oncology : official journal of the European Society for Medical Oncology / ESMO. 2008;19(10):1734-41.

50. Sun Q, Li F, Sun F, Niu J. Interleukin-8 is a prognostic indicator in human hilar cholangiocarcinoma. Int J Clin Exp Pathol. 2015;8(7):8376-84.

51. Lowe PR, Galley HF, Abdel-Fattah A, Webster NR. Influence of interleukin-10 polymorphisms on interleukin-10 expression and survival in critically ill patients. Critical care medicine. 2003;31(1):34-8.

52. Thepmalee C, Panya A, Junking M, Chieochansin T, Yenchitsomanus PT. Inhibition of IL-10 and TGF-β receptors on dendritic cells enhances activation of effector T-cells to kill cholangiocarcinoma cells. Human vaccines & immunotherapeutics. 2018;14(6):1423-31.

53. Lurje I, Czigany Z, Bednarsch J, Gaisa NT, Dahl E, Knüchel-Clarke R, et al. Genetic variant of CXCR1 (rs2234671) associates with clinical outcome in perihilar cholangiocarcinoma. Liver cancer. 2022:in press.

54. Alfaro C, Teijeira A, Oñate C, Pérez G, Sanmamed MF, Andueza MP, et al. Tumor-Produced Interleukin-8 Attracts Human Myeloid-Derived Suppressor Cells and Elicits Extrusion of Neutrophil Extracellular Traps (NETs). Clinical cancer research : an official journal of the American Association for Cancer Research. 2016;22(15):3924-36.

55. Tanimoto K, Yoshiga K, Eguchi H, Kaneyasu M, Ukon K, Kumazaki T, et al. Hypoxia-inducible factor-1alpha polymorphisms associated with enhanced transactivation capacity, implying clinical significance. Carcinogenesis. 2003;24(11):1779-83.

56. Noman MZ, Desantis G, Janji B, Hasmim M, Karray S, Dessen P, et al. PD-L1 is a novel direct target of HIF-1α, and its blockade under hypoxia enhanced MDSC-mediated T cell activation. J Exp Med. 2014;211(5):781-90.

57. Corzo CA, Condamine T, Lu L, Cotter MJ, Youn JI, Cheng P, et al. HIF-1α regulates function and differentiation of myeloid-derived suppressor cells in the tumor microenvironment. J Exp Med. 2010;207(11):2439-53.

58. Zhang X, Miao X, Tan W, Ning B, Liu Z, Hong Y, et al. Identification of functional genetic variants in cyclooxygenase-2 and their association with risk of esophageal cancer. Gastroenterology. 2005;129(2):565-76.

59. Han C, Leng J, Demetris AJ, Wu T. Cyclooxygenase-2 promotes human cholangiocarcinoma growth: evidence for cyclooxygenase-2-independent mechanism in celecoxib-mediated induction of p21waf1/cip1 and p27kip1 and cell cycle arrest. Cancer research. 2004;64(4):1369-76.

60. Miao J, Lu X, Hu Y, Piao C, Wu X, Liu X, et al. Prostaglandin E(2) and PD-1 mediated inhibition of antitumor CTL responses in the human tumor microenvironment. Oncotarget. 2017;8(52):89802-10.

61. Papafili A, Hill MR, Brull DJ, McAnulty RJ, Marshall RP, Humphries SE, et al. Common promoter variant in cyclooxygenase-2 represses gene expression: evidence of role in acute-phase inflammatory response. Arteriosclerosis, thrombosis, and vascular biology. 2002;22(10):1631-6.
